# Erdheim‐Chester disease progression from miliary pulmonary nodules to large tumours

**DOI:** 10.1002/rcr2.475

**Published:** 2019-08-23

**Authors:** Jun Shiihara, Hiromitsu Ohta, Satoshi Ikeda, Tomohisa Baba, Koji Okudera, Takashi Ogura

**Affiliations:** ^1^ Department of Respiratory Medicine Kanagawa Cardiovascular and Respiratory Center Kanagawa Japan; ^2^ Department of Respiratory Medicine Jichi Medical University Saitama Medical Center Saitama Japan; ^3^ Department of Pathology Yokohama City Graduate University School of Medicine Kanagawa Japan

**Keywords:** Erdheim‐Chester disease, Langerhans cell histiocytosis, miliary nodules, random distribution

## Abstract

Erdheim‐Chester disease (ECD), a rare form of non‐Langerhans cell histiocytosis, affects long bones, the retroperitoneal region, and the central nervous, cardiovascular, and pulmonary systems. Most patients with ECD show interlobular septal thickening, centrilobular micronodules, and ground glass opacities on chest computed tomography (CT). We encountered a 66‐year‐old man with ECD who presented at first visit with randomly distributed multiple pulmonary nodules and who then developed large tumour shadows, observed on chest CT. To our knowledge, this random distribution pattern of multiple pulmonary nodules has not previously been reported.

## Introduction

Erdheim‐Chester disease (ECD) is a rare form of non‐Langerhans cell histiocytosis (LCH) 1. To date, only several hundred cases have been reported. ECD is a haematopoietic neoplasm due to clonal proliferation of myeloid progenitor cells. Most patients with ECD harbour somatic mutation in the mitogen‐activated protein kinase signalling pathway, half of which involve a v‐raf murine sarcoma viral oncogene homolog B1 (BRAF) V600E mutation 2. ECD affects the long bones, the retroperitoneum, heart, lungs, central nervous system, skin, pituitary glands, and the orbit. The most common clinical manifestations of ECD are bone pain, diabetes insipidus, and neurological symptoms, and diagnosis of ECD is based on a pathological evaluation of the tissue involved. One pathological feature of ECD is infiltration of foamy histiocytes with inflammatory cells and multinuclear giant cells. These histiocytes are positive for vimentin, surface CD14, CD68, and S‐100 protein, and are negative for CD1a or langerin. CD1a and langerin are expressed in histiocytes of LCH 3. Radiological findings of ECD are variable. Bilateral and symmetric osteosclerosis of the diaphysis of the long bone is often detected using bone scintigraphy. Interlobular septal thickening, centrilobular nodular nodules, and ground glass opacities are frequently observed on chest computed tomography (CT).

We encountered a patient with ECD whose chest CT initially showed diffuse small randomly distributed nodules, resembling miliary tuberculosis. Within 6 months, bilateral tumour shadows had developed. It is rare to encounter a patient with ECD who presents on chest CT with diffuse multiple randomly distributed nodules; however, it is important to differentiate ECD from other diseases such as miliary tuberculosis, lung metastasis, and sarcoidosis.

## Case Report

**Figure 1:**
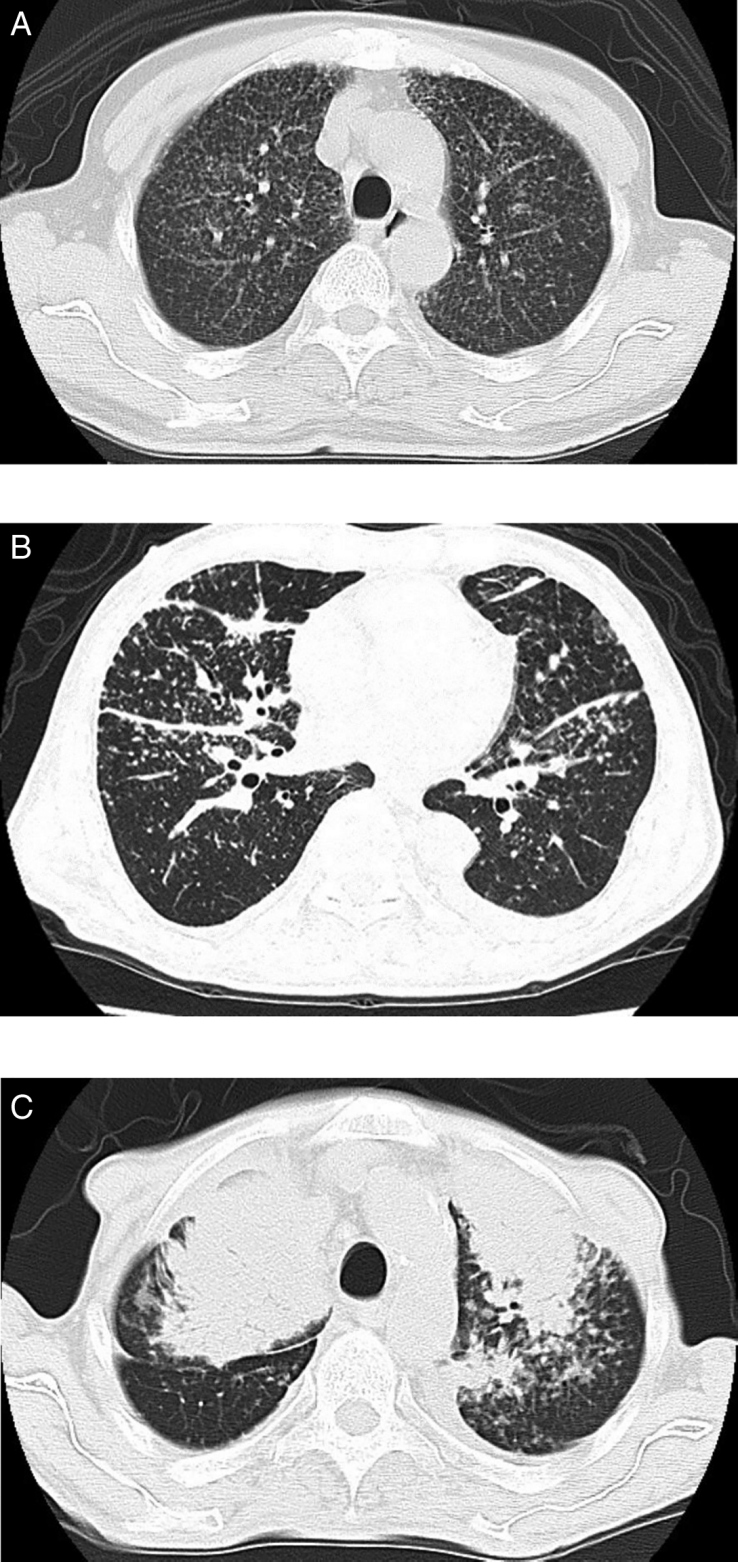
(A) High‐resolution computed tomography of the lung showed small diffuse nodules, randomly distributed throughout lung fields. (B) Chest computed tomography (CT) taken a few months after the initial CT showed thickening of interlobular septa and interlobar fissures, as well as small nodules. A small amount of bilateral pleural effusion was also observed. (C) Chest CT showed several large tumours in the bilateral lung region, in addition to small nodules. Pleural effusion is observed in the left thoracic cavity.

A 66‐year‐old man was admitted to hospital for further investigations concerning abnormal shadows seen on plain chest radiographs. He was asymptomatic, and his lung auscultation was normal. High‐resolution computed tomography (HRCT) of the lung showed reticular pattern and randomly distributed miliary nodules, and miliary tuberculosis or metastatic lung tumour was suspected (Fig. 1A). Thickening of interlobular septa and interlobar fissures had also appeared on a chest CT obtained a few months after the initial CT (Fig. 1B). Serum tumour marker and angiotensin‐converting enzyme levels were within a normal range, and the interferon‐γ release assay test was negative. Acid‐fast bacteria were not cultured and were not detected through polymerase chain reaction tests of the sputum, gastric juices, and bone marrow aspirate. A positron‐emission tomography scan revealed no significant uptake of fluorodeoxyglucose in the nodules. No definitive diagnosis was obtained from the transbronchial lung biopsy specimens. He was clinically diagnosed with interstitial pneumonia and miliary tuberculosis. He received methylprednisolone pulse therapy, which was then followed with prednisolone administered orally. Anti‐tuberculous therapy with isoniazid, rifampicin, pyrazinamide, and ethambutol was also initiated. His radiological findings gradually improved, and oral prednisolone therapy was decreased gradually and ended after 5 months. Anti‐tuberculous therapy continued for 6 months. However, 3 months following the discontinuation of anti‐tuberculous therapy, the shadows seen on chest CT had worsened, and large tumour shadows had formed in the bilateral upper lobe (Fig. 1C). He complained of dyspnoea and low‐grade fever, and steroid therapy was administered. However, radiological examinations showed little improvement and he was transferred to our hospital.

**Figure 2:**
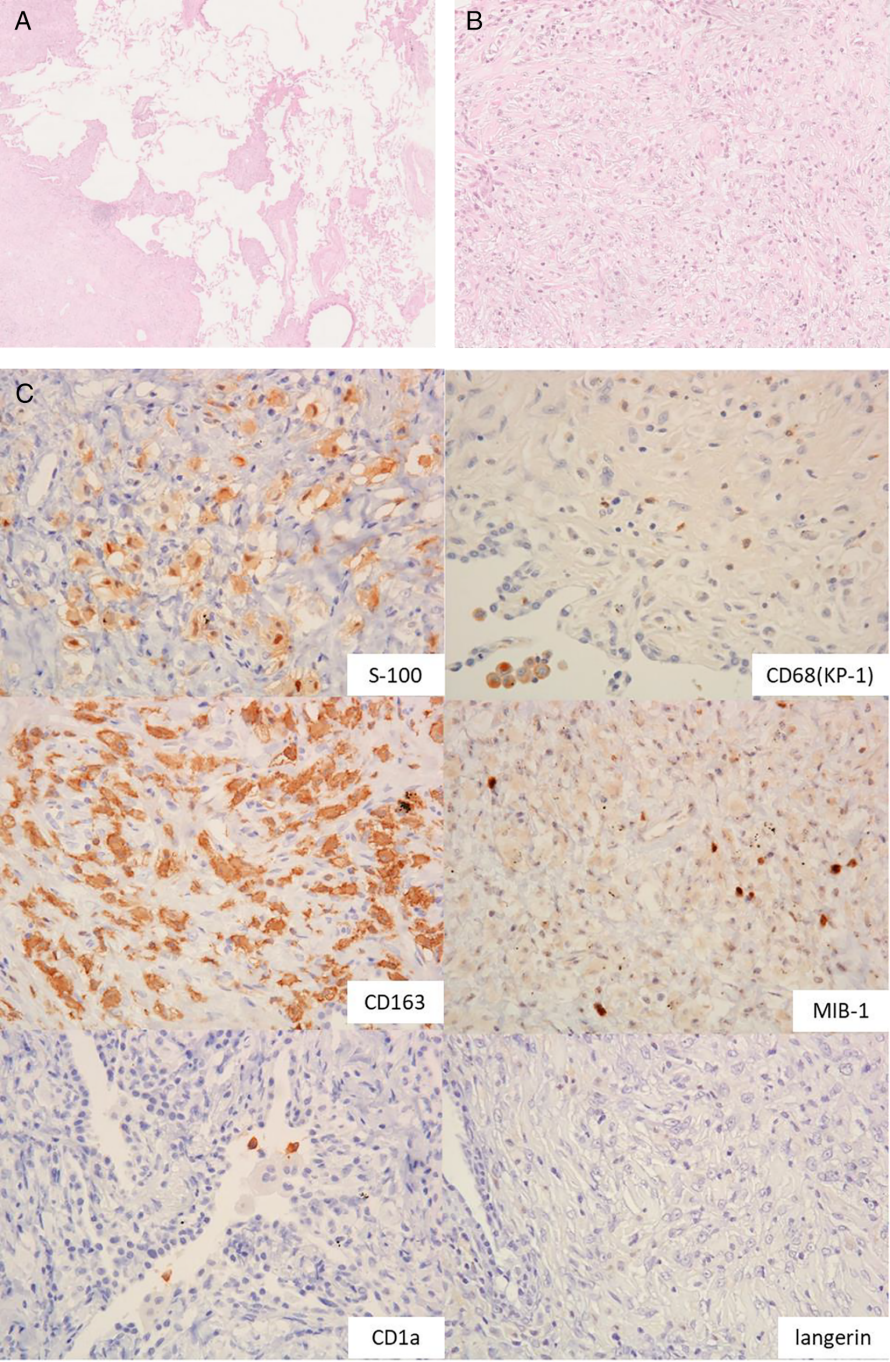
(A) Haematoxylin–eosin stained biopsy section revealing thickening of the interlobular septa and bronchovascular bundles and dense fibrosis in the pleura, interlobular septa, and peribronchovascular interstitium (low power, ×25). (B) Infiltration of large foamy histiocytes was observed in these fibrotic lesions (high power, ×200). (C) Immunohistochemical stains on lung biopsy. S‐100 and CD163 immunostaining showed positive staining of foamy histiocytes. CD68 and MIB‐1 immunostaining showing a weak positive stain. CD1a and langerin immunostaining showed negative staining.

To determine a definitive diagnosis, video‐assisted thoracoscopic biopsy of the right middle lobe was performed. Histological examination of the biopsy specimen revealed thickening of the interlobular septa and bronchovascular bundles. Severe fibrosis was found in the pleura, interlobular septa, and in the peribronchovascular interstitium. Proliferation of large foamy histiocytes was observed in these fibrotic lesions (Fig. 2A, B), which were characterized with constricted cell nuclei and acidophilic cytoplasm. No sarcoid type granuloma was found. Cells with coffee bean‐shaped nuclei and eosinophil infiltration, indicative of LCH, and phagocytosis of red blood cells were not identified. The large foamy histiocytes stained positive for S‐100 protein, CD68, CD163, and MIB‐1 (Fig. 2C). CD1a and langerin were negative in the histiocytes. Electron microscopy revealed no Birbeck granules in the cytoplasm. Bone scintigraphy showed bilateral and symmetrical 99mTc‐hydroxymethylene diphosphonate accumulations in the femur, tibia, and humerus. Plain radiographs of these bones showed osteosclerotic change. Magnetic resonance imaging of the brain showed a pituitary gland tumour and a thickening of the dura mater, enhanced with gadolinium. Based on these findings, we diagnosed ECD with bone, brain, and lung lesions. After confirming the ECD diagnosis, cyclosporin was added to the steroid therapy. He was temporarily relieved of his symptoms; however, he deteriorated gradually and died almost 12 months later.

## Discussion

Previous several studies have reported radiological findings of ECD on chest CT 4, 5, 6, 7. These studies have reported that between 50% and 90% of patients with ECD present with pulmonary involvement of the pleura, the lung parenchyma, or both. Recently, Toya et al. conducted a nationwide survey to collect detailed data of 44 patients with ECD, including our case, in Japan; and they reported pulmonary involvement was observed in 34.1% of the patients 8.

Because pulmonary involvement is sometimes asymptomatic and laboratory data are also often non‐specific, a diagnosis can be extremely difficult in cases such as the one we present here, in which there were neither symptoms accompanying the extrapulmonary lesion nor abnormalities in laboratory data (e.g. urine electrolytes or urine osmolality abnormalities, accompanying diabetes insipidus). Indeed, this patient was misdiagnosed as having miliary tuberculosis. A surgical lung biopsy was undertaken because of the unusual clinical course, and he was finally diagnosed with ECD.

According to studies regarding the radiological findings of ECD on chest CT, interlobular septal thickening (32%–69%) and pulmonary nodules (21%–62%) are the most frequent findings 4, 5, 6. Ground glass opacities (12%–36%), lung cysts (6%–12.5%), and consolidation (7.5%–9%) have also been observed (Table 1). Although most previous case reports and studies showed that a common distribution of pulmonary nodules on chest CT form a centrilobular pattern, Mirmomen et al. [6] found a subpleural pattern to be the most common pattern observed in their study, with nodules located in subpleural regions (36%), nodules in lung parenchyma (13%) that were centrilobular in distribution, and nodules in both regions (13%). Mirmomen et al. stated that it was difficult to determine an accurate distribution of nodules using older CT with thicker slices. For our patient, the HRCT showed a random distribution of small diffuse nodules, that is, the nodules were distributed in the interlobular septal, peribronchovascular, in addition to the centrilobular and subpleural region. These radiological findings corresponded with the pathological findings. Gradually, these small nodules progressed to large tumours. ECD is a progressive disease, in which histiocytes proliferate and form xanthogranulomatous lesions in a multicentric manner. Adjacent lesions may fuse together during expanding of the lesions.

**Table 1:** Radiological findings of ECD on chest CT.

Reference	Pulmonary involvement (%)	Interlobular septal thickening (%)	Pulmonary nodules	GGO (%)	Others
Mirmomen et al. 6	90	69	Centrilobular and subpleural (62%)	36	Airway thickening (13%)
Arnaud et al. 4	53	32	Centrilobular (21%)	12	Thickening of interlobar fissure (12%)
					Consolidation (9%)
					Cysts (6%)
					Microcystic lesions (6%)
Brun et al. 5	55	52.50	Centrilobular (22.5%)	20	Subpleural thickening along the fissure (32.5%) cysts (12.5%)
					Consolidation (7.5%)
					Microcystic lesions (7.5%)

GGO, ground glass opacities.

In symptomatic patients with ECD who have a BRAF V600E mutation, vemurafenib, a BRAF inhibitor, has been recommended for initial therapy 9. For those who do not have a BRAF V600E mutation, interferon‐alpha has been recommended 10. At the time of treatment for our patient, standard therapy had not been established, and he was treated with steroid and cyclosporin, which were not effective.

In this case report, our patient initially presented with a pattern of multiple randomly distributed small lung nodules, as observed on chest CT, which then progressed to large tumours. This type of disease progression has not previously been reported.

### Disclosure Statement

Appropriate written informed consent was obtained for publication of this case report and accompanying images.
